# Understanding Cluster Headache Using Magnetic Resonance Imaging

**DOI:** 10.3389/fneur.2020.00535

**Published:** 2020-06-30

**Authors:** Stefania Ferraro, Anna Nigri, Greta Demichelis, Chiara Pinardi, Luisa Chiapparini, Luca Giani, Alberto Proietti Cecchini, Massimo Leone

**Affiliations:** ^1^Neuroradiology Department, Fondazione IRCCS Istituto Neurologico Carlo Besta, Milan, Italy; ^2^Neurology Department, Fondazione IRCCS Istituto Neurologico Carlo Besta, Milan, Italy

**Keywords:** cluster headache, resting-state fMRI, structural MRI, PET, DTI, hypothalamus

## Abstract

Cluster headache is an excruciating pain syndrome characterized by unilateral head pain attacks, lasting between 15 and 180 min, accompanied by marked ipsilateral cranial autonomic symptoms, such as lacrimation and conjunctival injection. Despite important insights provided by neuroimaging studies and deep brain stimulation findings, the pathophysiology of cluster headache and its pathways of chronicization are still elusive. In this mini-review, we will provide an overview of the functional and structural neuroimaging studies in episodic and chronic cluster headache conditions conducted to clarify the underlying pathophysiology.

## Introduction

The distinctive clinical characteristic of cluster headache (CH), in particular the recurrence of excruciating unilateral attacks accompanied by marked ipsilateral cranial autonomic symptoms in periods separated by the spontaneous remission (1), suggested specific brain networks involved in seasonal adaptation (2) to have a role in the pathophysiology of this disorder. Neuroimaging can track these functional and anatomical changes (3), irrespective if they are the cause of the disease or represent a brain adaptation/maladaptation to the painful condition.

Brain networks involved in different phases of CH, namely, the *in-bout* (out of attacks and during attacks) and *out-of-bout* phases, will be presented. We will describe and discuss resting-state functional magnetic resonance imaging (rs-fMRI), positron emission tomography (PET) and single-photon emission computed tomography (SPECT), structural MRI, and diffusion tensor imaging (DTI) studies. Due to the recognized importance of the hypothalamus in CH pathophysiology, first, we will present studies investigating the hypothalamic/midbrain tegmentum. Then, we will describe MRI/PE/SPECT studies focusing on other cerebral areas and DTI investigations.

## Search and Selection of Studies

We searched electronic databases PubMed and Google Scholar for articles published in English between January 1996 and December 2019 (see Tables 1, 2). The search terms were: (“cluster headache”) AND (“functional magnetic resonance imaging” OR “fMRI” OR “functional connectivity”); (“cluster headache”) AND (“positron emission tomography” OR “PET” OR “single photon emission computed tomography” OR “SPECT” OR “cerebral blood flow”); (“cluster headache”) AND (“gray matter” OR “voxel based morphometry” OR “VBM” OR “cortical thickness”); (“cluster headache”) AND (“white matter” OR “diffusion tensor imaging” OR “DTI” OR “tractography”). We did not consider reviews and conference abstracts.

**Table 1:** List of metabolic and functional studies on cluster headache.

**Author(s)**	**Participants**	**Diagnosis: in/out of bout episodic, chronic**	**Whole brain/ROI analysis**	**Method**	**ROI coordinates**	**Aim/Hypothesis**	**Main results**
Hseih et al. (4)	4 in-eCH, 3 out-eCH	in-eCH, out-eCH	Whole brain	[15(O)] Butanol-PET	-	To investigate the central processing of CH attacks provoked by sublingual NTG in in-eCH and out-eCH.	During CH attacks, decreased rCBF in prefrontal, posterior parietal and occipito-temporal cortex, while increased rCBF in right and rostro-caudal anterior cingulate cortex, temporo-polar cortex, supplementary motor area, bilaterally motor and premotor areas, opercular region, insula, putamen, and lateral inferior frontal cortex.
Di Piero et al. (5)	7 out-eCH, 12 HC	out-eCH	Whole brain	Xe-133-SPECT	-	To investigate the different patterns of activation of the structures involved in tonic pain perception in out-eCH patients.	Decreased CBF in controlateral primary sensory motor cortex and thalamus controlateral to pain side.
May et al. (3)	9 cCH, 8 out-eCH	out-eCH, cCH	Whole brain	${\text{H}}_{2}^{15}$O-PET	-	To investigate changes in rCBF in cCH and out-eCH patients.	During CH attacks in cCH, significant activation was found in the ipsilateral inferior hypothalamic gray area (SPM MNI [−2, −18, −8]) with an additional increased rCBF in the thalamus, anterior cingulate cortex, and bilaterally in the insulae.
May et al. (6)	9 cCH, 8 out-eCH	out-eCH, cCH	Whole brain	${\text{H}}_{2}^{15}$O-PET	-	To investigate the NTG-induced CH attacks in cCH and out-eCH patients.	Significant activations in acute pain state in the left insula and right inferior frontal cortex, around major basal vessels, and in the left (ipsilateral to the pain) hypothalamic gray area.
Sprenger et al. (7)	1 cCH	cCH	Whole brain	${\text{H}}_{2}^{15}$O-PET	-	To investigate hypothalamic activation during a spontaneous CH attack in a cCH patient.	Increased activation in the ipsilateral inferior hypothalamus. Increased rCBF in the contralateral anterior cingulate cortex and the medial thalamus.
Sprenger et al. (8)	6 in-eCH, 1 cCH, 8 HC	in-eCH, cCH	Whole brain	[^11^C] DPN-PET	-	To investigate if the pathophysiology of CH may relate to opioidergic dysfunction in biologic clock circuitries.	Reduced opioid receptor binding in the pineal gland in CH patient.
Sprenger et al. (9)	11 in-eCH (retested during out of bout), 11 HC	in-eCH, out-eCH	Whole brain	FDG-PET	-	To investigate alteration of brain metabolism in eCH patients in bout and out of bout.	eCH vs. HC showed a decreased metabolism in the prefrontal and orbitofrontal cortex and an increased FDG metabolism in the parietal lobe and postcentral gyrus. in-eCH vs. out-eCH presented increased metabolism in the anterior cingulate cortex, posterior cingulate cortex, orbitofrontal cortex including nucleus accumbens, ventrolateral and dorsolateral prefrontal cortex, and temporal cortex, while decreased metabolism in the bilateral cerebellopontine area was reported.
Morelli et al. (10)	4 in-eCH	in-eCH	Whole brain	rs-fMRI: GLM	-	To investigate the difference in cerebral activation in in-eCH patients between the pain state of a spontaneous headache attack and the pain-free state.	Each in-eCH patient showed significant activation in the ipsilateral hypothalamic area (Tal [−5, −8, −1]) in the comparison of the pain with the pain-free state. A trend of activation was also detected in the prefrontal cortex, cingulate cortex, insula, cerebellum, thalamus, and basal ganglia.
Rocca et al. (11)	13 out-eCH, 15 HC	out-eCH	Hypothalamus	rs-fMRI: SBA, ICA	5-mm sphere MNI [±2, −18, −8]^*^	To investigate thebrain resting-state networks abnormalities in eCH.	Out-eCH, compared to HC, presented increased FC in the thalamus and the hypothalamus and decreased fluctuations within primary visual and sensorimotor networks.
Morelli et al. (12)	1 cCH (pain state, pain-free state after 6 mg sumatriptan administration)	cCH	Whole brain	rs-fMRI: GLM	-	To investigate fMRI findings in a cCH patient during pain and pain-free state.	Significant activation in the ipsilateral hypothalamic area (Tal [−3, −3,−8]) and brainstem regions (ipsilateral trigeminal root entry zone, bilateral red nucleus, ventral pons) were reported in the pain compared with the pain-free state. Trends of activations also in the prefrontal cortex, cingulate cortex, insula, cerebellum, thalamus, and basal ganglia.
Qiu et al. (13)	12 in-eCH, 12 HC	in-eCH	Hypothalamus	rs-fMRI: SBA	6-mm sphere Tal [2, −18, −8]	To investigate the FC alteration of the hypothalamus “during attack” and “out of attack.”	In-eCH, compared to HC, presented abnormal hypothalamic FC in the pain system during the spontaneous CH attacks. During CH attack, it extended beyond the pain system.
Yang et al. (14)	18 in-eCH, (retested during out of bout), 19 HC	in-eCH, out-eCH	Hypothalamus	rs-fMRI: SBA	4-mm sphere MNI [±4, −18, −8]	To investigate the resting-state FC of the hypothalamus in a group of eCH (scanned both in and out of bout) and compare them with HC.	eCH patients, in comparison to HC, presented hypothalamic FC changes with the medial frontal gyrus and occipital cuneus. in-eCH, compared to out-eCH, showed a decreased hypothalamic FC with the medial frontal gyrus, precuneus, and cerebellar areas. In all eCH, the number of annual bout correlated with hypothalamic FC in cerebellar regions.
Qiu et al. (15)	21 in-eCH, 21 HC	in-eCH	Hypothalamus	rs-fMRI: ICA	10-mm sphere MNI [±5, −18, −8]	To investigate if the FC of the hypothalamus and the salience network was altered during the remission state.	in-eCH, compared to HC, presented a decreased hypothalamus-salience network coactivation suggesting a possible role in the pathophysiology of the disorder.
Chou et al. (16)	17 in-eCH (retested during out of bout), 18 HC	in-eCH, out-eCH	Whole brain	rs-fMRI: ICA	-	To investigate the relationship between FC networks and the bout status.	All eCH (regardless of bout period), compared to HC, presented changes in FC in temporal, frontal, salience, default mode, somatosensory, dorsal attention, and visual networks. in-eCH, compared to out-eCH, presented FC changes in the frontal and dorsal attention networks. In all eCH, a lower FC in frontal network correlated with disease duration.
Faragò et al. (17)	17 out-eCH, 26 HC	out-eCH	Whole brain	rs-fMRI: ICA	-	To investigate the alteration of FC in out-eCH in order to find signatures of the increased excitability.	Out-eCH presented increased frequency-specific activity in the attention network ipsilateral to the headache side and in the contralateral cerebellar network.
Ferraro et al. (18)	17 cCH, 16 HC	cCH	Hypothalamus	rs-fMRI: SBA	Manual ROI: on coronal slices, 3 mm in the *y* direction, 7–10 mm posterior to the anterior commissure	To test the hypothesis of a defective FC between the posterior hypothalamus and diencephalic–mesencephalic regions in cCH.	cCH, compared to HC, showed increased FC between the ipsilateral posterior hypothalamus and a number of diencephalic–mesencephalic structures, comprising ventral tegmental area, dorsal nuclei of raphe, and bilateral substantia nigra, subthalamic nucleus, and red nucleus. No difference was found comparing the contralateral hypothalami.

*In this table, we reported the regions as defined in the original manuscripts. ROI coordinates were reported as [x, y, z], unless otherwise noted. CH, cluster headache; in-eCH, in-bout episodic CH; out-eCH, out-of-bout episodic CH; cCH, chronic CH; HC, healthy controls; ROI, region of interest; SPECT, single-photon emission computed tomography; PET, positron emission tomography; FDG, ^18^Fluoro-2-deoxy-D-glucose; NTG, nitroglycerin; [^11^C]DPN, [^11^C]diprenorphine; ${\text{H}}_{2}^{15}$O, 15O-labeled water; Xe-133, Xenon-133; CBF, cerebral blood flow; rCBF, regional cerebral blood flow; rs-fMRI, resting state functional magnetic resonance imaging; SBA, seed-based analysis; ICA, independent component analysis; FC, functional connectivity; Tal, Talairach coordinates; MNI, Montreal Neurological Institute. *(

* *), May et al. (19) coordinates*.

**Table 2:** List of structural studies on cluster headache.

**Author(s)**	**Participants**	**Diagnosis: in/out of bout episodic, chronic**	**Whole brain/ROI analysis**	**Method**	**ROI coordinates**	**Aim/Hypothesis**	**Main results**
May et al. (19)	25 CH (14 active headache, 11 headache-free state), 29 HC	eCH, cCH	Whole brain	sMRI: VBM; PET	-	To investigate structural and functional metabolic brain alterations between CH patients and HC.	Colocalization of an increase in GM (SPM MNI [−4, −16, −10]) and functional activation (SPM MNI [−2, −18, −8]) ipsilateral to the pain side in the inferior posterior hypothalamus was identified in CH patients compared to HC. This structural alteration was also present when comparing patients during active headache and headache-free state with HC.
Matharu (20)	66 eCH, 96 HC	ND	Whole brain and hypothalamus	sMRI: VBM	-	To investigate structural brain alterations between eCH and HC.	No significant changes in GM and WM.
Owen et al. (21)	1 cCH, 13 HC	cCH	DBS-targeted region (hypothalamus)	DTI: probabilistic tractography	6 mm posterior, 2 mm lateral and 8 mm below the mid-commissural point^*^	To investigate the structural connectivity of the posterior inferior hypothalamus in HC, using coordinates derived from a patient implanted with a DBS electrode.	In the HC, the seed of the DBS target coordinates was connected with the medial lemniscus, ipsilateral fronto-orbital cortex, reticular nucleus, superior cerebellar peduncle, cerebellar cortex.
Teepker et al. (22)	1 in-eCH, 6 out-eCH, 7 HC	in-eCH, out-eCH	Whole brain	DTI: diffusivity maps	-	To investigate microstructural alterations in patients with eCH vs. HC.	Bilateral brainstem, internal capsule, superior/inferior temporal region, frontal lobe, occipital lobe, and right thalamus and cerebellum showed an altered WM microstructure in eCH patients compared to HC.
Absinta et al. (23)	15 out-eCH, 19 HC	out-eCH	Whole brain	sMRI: VBM; DTI: diffusivity maps	-	To investigate if the patterns of regional GM and WM alterations in out-eCH are confined to the hypothalamus or tend to be more widespread to the central nervous system.	A decrease in GM volume in several cortical and subcortical regions, part of the so-called “pain-matrix network,” was reported in out-eCH patients vs. HC. A decrease in GM volume of left middle frontal gyrus significantly correlated with disease duration. No difference was found in WM between out-eCH and HC.
Seifert et al. (24)	12 out-eCH, 12 HC	out-eCH	Whole brain	sMRI: cortical thickness	-	To investigate cortical thickness abnormalities in out-eCH patients compared to HC. They expected changes in cortical thickness in pain-processing areas.	A reduction of cortical thickness in the angular gyrus and the precentral gyrus was shown in CH patients contralaterally to the headache side compared to HC. Cortical thickness in the primary sensory cortex correlated with disease duration.
Szabò et al. (25)	13 eCH, 16 HC	ND	Whole brain	DTI: diffusivity maps		To investigate WM microstructure in eCH patients with multiple diffusivity measures.	WM alterations in eCH compared to HC were found in frontal, parietal, temporal and occipital lobe, principally contralateral to the attack side.
Yang et al. (26)	49 in-eCH (12 retested during out bout period), 49 HC	in-eCH, out-eCH	Whole brain and hyphothalamus	sMRI: VBM, hyphotalamus (SVC)	MNI [−4, −16, −10]^**^	To investigate (1) if structural changes in in-CH patients are restricted to the hypothalamus or tend to be widespread to pain modulation regions; (2) longitudinal structural alterations of CH between in- and out-bout periods.	(1) A significant GM volume reduction in frontal pain modulation areas was reported in in-eCH and out-eCH patients compared with HC, while (2) a significant GM increase in the left anterior cingulate, insula, and fusiform gyrus was revealed in-eCH compared to out-eCH patients.
Chou et al. (27)	17 in-eCH (retested during out of bout), 17 HC	in-eCH, out-eCH	Whole brain for diffusivity maps, hypothalamus for tractography	DTI: diffusivity maps, probabilistic tractography	Hypothalamus (manual segmentation)	To investigate (1) WM alterations between in-eCH and out eCH and (2) the anatomical connections between the hypothalamus and brain areas with WM changes.	(1) Compared to HC, in-eCH showed significant differences in the right side in inferior and superior longitudinal fasciculus, anterior thalamic radiation. In out-eCH vs. HC, WM alterations were found in right inferior and superior longitudinal fasciculus, bilateral corpus callosum, and left cortico-spinal tract. Differences between in-eCH and out-eCH were present in the left cerebellum WM. (2) The ipsilateral hypothalamus showed projections with frontal and limbic areas and cerebellum.
Clelland et al. (28)	7 HC	-	DBS-targeted regions (midbrain tegmentum)	DTI: probabilistic tractography	From the midpoint of the AC-PC line [± 2, −3, −5]; [±2, −6, −8]; [±2.98, −3.53, −3.31]; [±4 mm from the 3rd ventricle wall, −2, −5]	To investigate the structural connectivity of efficacious DBS-targeted regions in HC in order to highlight anatomic connections that are involved in modulating CH attacks.	DBS target coordinates were located in the midbrain tegmentum gray matter. Common structural connections were found from DBS-targeted seeds to ipsilateral hypothalamus, ipsilateral reticular formation, and ipsilateral cerebellar cortex.
Naegel et al. (29)	46 out-eCH, 22 in-eCH, 23 cCH, 78 HC	in-eCH, out-eCH, cCH	Whole brain	sMRI: VBM	-	To investigate different GM change patterns corresponding to different stages of disease (in-eCH, out-eCH, cCH) in order to differentiate structural abnormalities associated with CH pathophysiology from changes related to the pain.	GM alterations (including also the temporal lobe, the hippocampus, the insular cortex, and the cerebellum) in the different stages of the disease were different for extension, location, and direction. Dynamic relation between pain vs. no-pain state was reported. No structural alterations in the hypothalamus were detected in CH patients compared to HC.
Akram et al. (30)	7 cCH	cCH	DBS-targeted regions (VTA)	-	DTI: probabilistic tractography	To investigate (1) the optimal DBS target in a sample of cCH and (2) structural connections pathway of this DBS target in responder patients.	(1) The target volume of responder DBS was located in the VTA (MNI [−4, −12, −8]), posterior to the hypothalamus in the ventral tegmentum; (2) in the responder group, this target showed a common pathway toward inferior-laterally to the amygdala and the temporal pole, anterosuperiorly to the prefrontal area and posteriorly to a dorsolateral position toward the trigeminal tract and nuclei.
Arkink et al. (31)	25 eCH, 27 cCH, 14 probable CH, 9 chronic paroxysmal hemicrania, 35 migraine, 48 HC	eCH, cCH	Whole brain and hypothalamus	sMRI: VBM, hypothalamus (SVC, manual segmentation)	MNI [(−12, 12), (6, −18), (0, −20)]	To investigate if (1) there are structural changes in the hypothalamus or other structural brain regions in eCH and cCH; (2) these changes are characteristic of CH or can also be found in other episodic headache disorders.	In comparison to HC, the anterior hypothalamus was enlarged in eCH, cCH, probable CH, and chronic paroxysmal hemicrania, but not in migraine. Widespread changes in pain modulation regions were reported in all patients with headache.
Kiraly et al. (32)	22 out-eCH, 94 HC	out-eCH	Subcortical nuclei	DTI: diffusivity maps (ROI analysis)	ROI from FSL MNI atlas	To investigate the GM alterations of the subcortical structures in out-eCH using diffusivity measures.	The subcortical gray nuclei microstructure was altered in eCH compared to HC in the bilateral amygdala, right caudate, and right pallidum.
Seijo-Fernandez et al. (33)	15 cCH, 11 HC	cCH	DBS-targeted region	DTI: deterministic and probabilistic tractography	4 mm lateral from the wall of the third ventricle, 2 mm behind and 5 mm below the intercommissural point	To investigate in HC subjects the structural connectivity of the mean target for DBS found in cCH patients.	In the HC, the structural connections between the stimulation target and posteriorly the cerebellar peduncle and the posterior mesencephalic tegmentum and anteriorly the frontal cortex and the forebrain were identified.
Giorgio et al. (34)	12 out-eCH, 13 migraine, 13 HC	out-eCH	Whole brain	sMRI: VBM; DTI: diffusivity maps; rs-fMRI: ICA, Network Analysis	-	To investigate structural and functional brain changes in out-CH compared to migraine patients and HC.	Out-eCH, compared to HC and migraine, showed decreased regional GM volume in the frontal cortex, higher short-range FC in networks involved in working memory and executive functions, while, when comparing them only to migraine, higher long-range FC in networks related to language processing were found. No differences in WM microstructure were reported.
Dantas et al. (35)	1 cCH	cCH	DBS-targeted region	DTI: deterministic tractography	1.5 mm lateral, 5 mm posterior, and 5 mm inferior relative to the midcommissural point	To investigate fiber tracts emerging from DBS target to explain the clinical effects of deep brain stimulation.	The fiber tracts projecting from the target region of DBS were the medial forebrain bundle, the dorsal longitudinal fasciculus, and the tracts connecting the hypothalamus to the brainstem.

*In this table, we reported the regions as defined in the original manuscripts. ROI coordinates were reported as [x, y, z], unless otherwise noted. CH, cluster headache; in-eCH, in-bout episodic CH; out-eCH, out-of-bout episodic CH; cCH, chronic CH; HC, healthy controls; ND, not defined; ROI, region of interest; sMRI, structural magnetic resonance imaging; PET, positron emission tomography; DTI, diffusion tensor imaging; rs-fMRI, resting state functional magnetic resonance imaging; DBS, deep brain stimulation; GM, gray matter; WM, white matter; FC, functional connectivity; VBM, voxel-based morphometry; SVC, small volume correction; ICA, independent component analysis; AC-PC, anterior commissure–posterior commissure line; MNI, Montreal Neurological Institute; *(

* *), Leone et al. (36) coordinates; *(

** *), May et al. (19) coordinates*.

## Neuroimaging Studies Investigating the Hypothalamus/Midbrain Tegmentum

### PET and rs-fMRI Studies

The circadian and circannual rhythmicity of attacks and neuroendocrinological findings pointed to hypothalamic involvement in the pathophysiology of the CH (2). In 1998, May et al., using PET, confirmed this hypothesis showing increased blood flow in the ipsilateral-to-the-pain posterior–inferior hypothalamus during nitroglycerin-induced attacks (3). This abnormal activity in the hypothalamus was considered to trigger the CH attacks because it was not observed in patients in *out-of-bout* phase who did not experience the attack under nitroglycerin (3). This seminal observation was confirmed by a subsequent PET study (6) and by a voxel-based morphometry study (19) showing hypothalamic volume abnormalities in CH patients (see the *Structural MRI studies* section). These results have opened the doors to hypothalamic deep brain stimulation (DBS) to successfully treat intractable chronic CH patients (36). Subsequently, the hypothesis of the hypothalamic involvement was supported by a single-case PET study (7) showing a metabolic activity during spontaneous attacks and by a ligand PET study showing opioidergic changes in the region identified by May et al. (3) in episodic CH patients during the *in-bout* phase (9).

More recently, the area reported as posterior–inferior hypothalamus by (3, 7, 19) has been suggested to localize in the midbrain tegmentum, possibly the ventral tegmental area (37, 38).

Despite this dispute, an fMRI study reported an evident activity in the ipsilateral-to-the pain hypothalamus during spontaneous attacks in a series of episodic CH patients (10). Importantly, this activity was not constrained by a region of interest approach centered on the results of the work of May et al. (3) but emerged using a whole-brain approach, reinforcing the hypothesis that the hypothalamus might play a role in CH. A paroxysmal activity during CH attacks in the red nuclei was also reported (12).

The observation that hypothalamic DBS in intractable chronic CH patients presents clinical effects after weeks of stimulation (39) and that it is not effective in terminating ongoing CH attacks (40) led us to hypothesize that the hypothalamus is a crucial region of a complex functional network that might disinhibit the hypothalamic–trigeminal pathway. In this new framework, the area reported by May et al. (3), hereafter “midbrain tegmentum” (37, 38), and the hypothalamus as such were investigated in a series of studies in CH patients using rs-fMRI to detect possible abnormalities in the functional connectivity (FC) of these regions.

The first study using rs-fMRI (11) showed that episodic CH patients have increased FC between the ipsilateral-to-the pain midbrain tegmentum and several regions known to be involved in pain processing, such as the anterior cingulate cortex, the bilateral secondary somatosensory cortex, the thalamus, and insula. Interestingly, abnormal FC was also observed between the midbrain tegmentum and striate and extra-striate visual regions, indicating the involvement of extra-pain-processing areas.

More recently, Qiu et al. (15) showed that episodic CH patients presented decreased bilateral midbrain tegmentum-salience network co-activation during the cluster period (*in-bout* phase). Previous works have shown that the dorsal anterior cingulate cortex and the fronto-insular cortex represent salient stimuli, such as hunger (41) and pain (42), and respond to emotional pain, such as during social rejection; in the seminal work of (43) these regions were appreciated with rs-fMRI as a robust functional network, namely, the salience network, also comprising subcortical structures such as thalamus, hypothalamus, and ventral tegmental area/substantia nigra. Seeley et al. (43) proposed that the relevant homeostatic stimuli, as sensory information, are integrated with visceral and autonomic functions, supporting a capital role of this network in pain processing. Based on this hypothesis (15), suggested that the bilateral midbrain tegmentum might play a role in the dysregulation of the salience network, in particular suggesting a defective pain control capable of generating CH attacks (15). The authors did not study CH patients *out-of-bout* phase: this does not allow one to make any inferences about the stability of this dysfunctional connectivity that might be dynamic (only during the *in-bout* phase) or a trait of CH patients. Notably, the same group showed that, in episodic CH patients investigated during attacks, the ipsilateral-to-the pain midbrain tegmentum presented abnormal dysfunctional connectivity with several cortical and subcortical areas (13). These areas comprised not only pain-processing regions but also extra-pain-processing areas. Notably, some of the identified areas (posterior cingulate cortex, inferior parietal lobule, ventral medial prefrontal cortex, and parahippocampal gyrus) belong to the default mode network.

Yang et al. (14) investigated for the first time the FC of the hypothalamus. They showed that, in accordance with its modulatory role, the hypothalamus is dynamically tuned, as it appears during the *in-bout* and the *out-of-bout* phases. During the *in-bout* phase, the ipsilateral-to-the pain hypothalamus presented, when compared to the *out-of-bout* phase, a decreased FC with the precuneus, a key region of the default mode network. This observation, with the results from the work of (13) during CH attacks, seems to confirm that dynamic alterations of the FC exist between the midbrain tegmentum/hypothalamus and regions belonging to the default mode network. Remarkably, the parasympathetic system is hypothesized to map onto the regions of the default mode network (44). Therefore, this dynamic dysregulation, present during the *in-bout* phase (13, 14), might indicate a parasympathetic dysfunction, possibly linked to the autonomic phenomena of CH. Further, Yang et al. (14) suggested that hypothalamic FC abnormalities in CH brain go beyond the pain matrix: when comparing patients *in-bout* vs. *out-of-bout* phase, the ipsilateral-to-the pain hypothalamus presented a decreased FC with the medial frontal gyrus and the cerebellar areas. These results seem to support dynamic alterations of hypothalamic FC in the disease. Moreover, the work of Yang et al. (14) showed that the annual bout frequency correlated significantly with the degree of FC between the hypothalamus and the cerebellar areas, suggesting that this might be an effect of the CH pathophysiology (14).

It is important to note that dysfunctional connectivity between the posterior hypothalamic regions and the midbrain areas was observed in chronic CH patients out of attacks (18). The authors showed an increased FC between the ipsilateral posterior hypothalamus and several diencephalic–mesencephalic structures as the ventral tegmental area, the dorsal nuclei of raphe, and the bilateral substantia nigra, the subthalamic nucleus, and the red nucleus. These results suggest a deranged FC of the hypothalamic–midbrain pathway in CH mainly involving structures that are part of (i.e., ventral tegmental area, substantia nigra) or modulate (dorsal nuclei of raphe, subthalamic nucleus) the midbrain dopaminergic systems. The latter may have a role in the chronicization of CH. Future studies should address the question if this abnormality is specific to chronic CH or it is already presented, with a lesser extent, in episodic CH.

As a whole, the above results show that the paroxysmal functional hyperactivity in the hypothalamus/midbrain tegmentum during induced and spontaneous CH attacks (3, 7, 12) is a dynamic process that appears to involve, in particular, FC changes between the midbrain tegmentum and regions belonging to the default mode network (13). This might suggest a paroxysmal activity of the parasympathethic system.

During *in-bout* and *out-of-bout* phases, the hypothalamus/midbrain tegmentum presents FC changes with different regions of the salience network (15) and the default mode network (14), suggesting, respectively, defective pain control and parasympathethic dysfunction. Moreover, it is essential to note that abnormal FC between the midbrain tegmentum and pain and extra-pain-processing regions is also observed in *out-of-bout* phase (11). This might indicate the presence of stable deranged connectivity between the hypothalamus/midbrain tegmentum and those areas. However, the observation of Yang et al. seems to indicate that further FC abnormalities between the hypothalamus and extra-pain-processing areas might superimpose on the already present FC alterations during the *out-of-bout* phase (14).

### Structural MRI Studies

The possible involvement of the region defined as inferior–posterior hypothalamus in the work of May et al. (3) during CH attacks was supported by a VBM study of the same group showing morphological alterations of this region in a relatively large cohort of episodic (*in-bout* and *out-of-bout phase*) or chronic CH patients (19).

Matharu in his PhD thesis (20) investigated a large sample of patients and analyzed the morphological data with updated software using also small volume correction in the region identified in the work of (3). Importantly, Matharu did not find volumetric abnormalities in this region and concluded that the previous VBM results (19) were false positive due to methodological limitations, possibly due to the susceptibility of the employed technique (VBM) to several confounders (45, 46).

In agreement, the VBM results from (3) were not replicated in subsequent morphological studies (23, 26, 47).

Despite these inconsistencies, a recent investigation showed an increased volume of the bilateral anterior hypothalamus of individuals with episodic (*out-of-bout*) and chronic CH but not in individuals with migraine (31). This study directly pointed to alterations of the suprachiasmatic nucleus, the site of the endogenous biological clock, and the paraventricular nucleus, both part of the anterior hypothalamus. Their abnormalities could explain the typical circadian rhythms of the recurrent attacks of CH, as well as some autonomic phenomena of the disease. These results confirm hypothalamic morphological alteration in episodic (in both *in-bout* and *out-of-bout*) and in chronic CH patients. It is important to note that possible dynamic morphological changes of the hypothalamus might have been underestimated due to the difficulties in investigating this relatively small structure with MRI.

## Neuroimaging Studies Investigating Pain-Processing Areas

### PET/SPECT and rs-fMRI Studies

The excruciating nature of pain in CH led us to hypothesize a possible deficient top-down modulation of antinociceptive circuits (9). In line with this hypothesis, several works have shown functional alterations of the pain-processing areas. The first PET study on CH dated back to 1996: Hsieh et al. (4) found increased regional blood flow in the main cortical regions involved in pain processing (anterior cingulate cortex, insula cortex, and operculum) during induced CH attacks.

Decreased cerebral blood flow activity in controlateral primary sensory-motor cortex and thalamus was also described during the cold pressor test (5).

Metabolic alterations of several brain areas, comprising regions involved in pain processing, were also shown in episodic CH: during the *in-bout* phase, compared to the *out-of-bout* phase, increased metabolism was observed in the anterior and posterior cingulate cortex, prefrontal cortex, insula, thalamus, and temporal cortex, while a decreased metabolism in the cerebellopontine areas (9).

Alterations of the pain-processing pathways were also reported in rs-fMRI studies. Rocca et al. (11) reported reduced FC in the sensorimotor network in a group of episodic CH patients *out-of-bout*. Affected brain areas comprised the primary and secondary somatosensory area, the supplementary motor area, and the anterior cingulate cortex (11). These regions play a role in sensory discrimination and affective-cognitive processes evoked by painful conditions. Notably, the anterior cingulate cortex is part of the salience network (43, 48): alteration of the FC in this region reinforces the hypothesis of a strong involvement of this circuit in CH pathophysiology (15). These abnormalities were observed in *out-of-bout* phase, indicating stable brain alterations. Importantly, disease duration was negatively correlated with the strength of FC in the primary sensory-motor cortex, indicating that prolonged and severe painful condition may have induced those alterations.

FC alterations in the sensorimotor network were confirmed in another study in episodic CH, which reported abnormalities also in regions of the salience network (16). This study found no differences between the *in-bout* and *out-of-bout* phase, suggesting that the functional alterations in the sensorimotor and salience network could be a trait marker of CH.

As a whole, a dynamic dysregulation or adaptation in networks involved in pain processing and modulation of the parasympathetic activity, as suggested by functional abnormalities in regions of the default mode network (i.e., anterior cingulate cortex), seems to characterize CH patients. These functional alterations may represent a derangement of descending pain processing and autonomic pathways.

### Structural MRI Studies

In patients with episodic CH during *out-of-bout* phase, volumetric alterations of the regions involved in pain processing such as thalamus, caudate nucleus, posterior cingulate cortex, prefrontal cortex, sensorimotor cortex, parietal cortex, insula, and middle temporal cortex have been reported (23, 34).

Notably, the chronic CH condition, compared to episodic CH, seems to be characterized by decreased gray matter in different regions of the pain matrix, i.e., anterior insula, cingulate cortex, secondary somatosensory cortex, hippocampus, left temporal lobe, and an increased gray matter in primary somatosensory cortex and supplementary motor cortex (47).

## Neuroimaging Studies Investigating other Cortical and Subcortical Areas

### PET and rs-fMRI Studies

In a large group of episodic CH patients, functional alterations in the default mode network have been observed in both *in-bout* and *out-of-bout* phases of CH (16). This suggests that the default mode network is dysfunctional in episodic CH patients, possibly as a trait marker of CH.

Rocca et al. (11) and Chou et al. (16) consistently reported alterations in the FC of the visual network in CH patients: these abnormalities might be linked to photophobia and retro-orbital pain, frequently observed in CH (49). These abnormalities were observed in *in-bout* (16) and *out-of-bout* phase (11, 16) and were negatively correlated to disease duration, suggesting that they might be the consequence of a prolonged and severe pain condition.

In agreement with widespread alterations, dysfunctional connectivity within the attention network (in the ipsilateral superior frontal gyrus and medial frontal cortex) and the cerebellar network was observed (17) in episodic CH patients in the *out-of-bout* phase. Abnormal FC in temporal and visual networks irrespective of the illness phase was also present.

### Structural MRI Studies

In VBM studies, volumetric gray matter alterations of the visual cortex (cuneus and occipital fusiform gyrus) have been observed (23, 34).

Volumetric alterations were observed by Naegel et al. in the temporal lobe, the hippocampus, the insular cortex, and the cerebellum in CH; (47) the location and direction of gray matter alterations varied according to the state of disease as well as to pain state (pain vs. no-pain). These dynamic changes may provide an explanation of the non-homogeneous results in previous VBM studies in pain.

## DTI

DTI is an advanced MRI technique measuring water molecule diffusion: it allows one to study the integrity and architecture of the tissues through several parameters, such as fractional anisotropy or mean, axial, and radial diffusivity (50). These quantitative indices are sensible to microstructural brain tissue properties such as axon diameter, density and orientations, axon myelination, and membrane permeability (51). Studying the principal diffusion direction, it is also possible to reconstruct through tractography continuous white matter pathways of significant clusters of parallel axons (51).

In the last decade, DTI has been applied in a CH population using both quantitative indices, employing the abovementioned parameters, or tractography.

The studies using the quantitative approach showed different results in episodic CH patients. While some groups did not find any significant differences in diffusion parameters (23, 34) in the *out-of-bout* phase, others reported patterns of stable alterations (i.e., in both *in-bout* and *out-of-bout* phase) in the white matter, mainly localized in frontal and limbic lobes (27). Importantly, these regions were shown to present anatomical connections with the hypothalamus when using probabilistic tractography (27).

Gray matter microstructure abnormalities were also reported in several subcortical structures, in particular in the right amygdala, caudate, and globus pallidum (32).

Interestingly, an increased axial diffusivity in the left cerebellar white matter when comparing patients in the *in-bout* and *out-of-bout* phase was also reported (27).

Other quantitative studies, focused on relatively small cohorts of episodic CH patients, highlighted very widespread diffusion microstructural abnormalities in different white matter regions, encompassing mainly temporal, frontal, occipital, and cerebellar regions (22, 25).

Tractography studies were mainly focused on the anatomical connections of successful DBS targets in chronic CH patients in the effort to reveal the anatomical network responsible for the amelioration of the disorder. These studies confirmed the relevance, in CH pathophysiology, of the target-brainstem projections. Notably, different DBS targets were considered: inferior–posterior hypothalamus (21), midbrain tegmentum (28, 33), and ventral tegmental area (35, 52). In chronic CH patients, the structural connectivity of these regions also showed extended and relevant connections to pain-related areas, supporting the hypothesis of large pain matrix modulating CH attacks.

In particular, important anatomical connections were highlighted between the hypothalamus and the midbrain tegmentum, including the medial lemniscus, the dorsal longitudinal and mamillo-tegmental fasciculi, the fronto-orbital cortex, the reticular shape, and the cerebellar cortex (21, 28, 33, 35).

In the case of DBS target stimulation in the VTA, further connections with the temporal cortex and brainstem areas in the proximity of the parabrachial nuclei, nucleus of the solitary tract, periaqueductal gray, and ending in the region of the trigeminal nucleus and tract and the superior salivatory nucleus were described (52). Despite these very interesting results, the small sample combined with heterogeneous inclusion criteria, the very different MRI sequences settings, and the different diffusion indices selection complicate the direct comparison between these works and may cause the observed discrepancy in particular in the results of quantitative diffusion analysis.

## Conclusion

Notwithstanding the notable number of neuroimaging studies in CH, we are still far from fully understanding the brain mechanisms of this disorder. However, some, although tentative and not conclusive considerations, can be done. First of all, CH patients seem to present widespread FC and anatomical abnormalities across multiple networks and multiple cortical and subcortical areas, not only confined in regions involved in pain processing. This suggests that the CH brain is functionally and morphologically reorganized in a maladaptive or adaptive way. In particular, functional and anatomical abnormalities of cortical and subcortical areas involved in pain processing are consistently reported. In this perspective, the salience network seems to play a prominent role in CH pathophysiology: the here reviewed studies suggest that regions of this network presents a relatively stable functional alteration during the *in-bout* and the *out-of-bout* conditions (16). One can speculate that dysfunctional connectivity in the salience network might be the neural “tract” of the disease. Notably, alteration of the salience network suggests that CH patients present a dysfunctional ability to elaborate salient stimuli. In this regard, abnormalities of this network were observed in other chronic pain conditions, such as diabetic neuropathy (53), headache, (54, 55), and irritable bowel syndrome (56). This might indicate that the abnormalities in the salience network might predispose the chronification of CH. Importantly, disruption of the salience network has been reported in several neuropsychiatric conditions such as autism (57), schizophrenia (58), and addiction (59). Therefore, the observed alterations seem not to be specific to CH. Future studies should assess the role of this network in CH pathophysiology. Moreover, regions belonging to the default mode network seem to be abnormal in CH, irrespective of the phase (16). The parasympathetic system maps onto the default mode network (44), and this raises the possibility that default mode network alterations are consequences of the CH attacks. The default mode network plays a role in integrating sensory-visceromotor processing, self-referential activity, and recalling of previous experience (60). This might indicate that CH patients suffer a disturbance in the social–emotional spheres.

Second, supporting the above results, studies investigating the hypothalamic/midbrain tegmentum FC suggest a modulatory role of these structures within the salience network and the regions of the default mode network (15, 16).

Third, the abnormal FC between the hypothalamus and the midbrain dopaminergic system (in particular the ventral tegmental area) in chronic CH patients (18) suggests that the possible pathways of chronicization pass through the mesocorticolimbic system also in CH. It is now accepted that the midbrain dopaminergic system is also stimulated by aversive stimuli such as pain (61), and the nucleus accumbens, a key component of the mesocorticolimbic system receiving direct projections from the ventral tegmental area, seems to be involved in the chronicization of pain in humans (62–64). This possibility in CH is also fostered by the recent proposal of the ventral tegmental area as the main target of DBS (65) and by the observation that long-term DBS can revert chronic to episodic CH (66). It is important to note, however, that inhibition and facilitation of pain mechanisms were also suggested to be at the basis of the chronicization of the disease, as indicated by gray matter reorganization accordingly with the different pain states, possibly supported by highly dynamic changes in nociceptive and anti-nociceptive networks (29). Also, DBS in CH patients induces blood flow changes in the anterior cingulate, insula, and frontal lobe involved in pain chronicization (67).

Future studies should assess the validity of the above hypotheses clarifying the role of the hypothalamus/midbrain tegmentum in CH and chronic CH pathophysiology (68). Moreover, it would be of great interest to determine if the observed abnormalities in functional and anatomical networks are specific to CH or represent an unspecific response to pain.

## Author Contributions

SF, AN, GD, CP, LC, LG, and AP went through on literature and collected the articles. SF, AN, and ML wrote the manuscript. ML supervised all the steps of this minireview. All authors contributed to the article and approved the submitted version.

## Conflict of Interest

The authors declare that the research was conducted in the absence of any commercial or financial relationships that could be construed as a potential conflict of interest.
